# Hemophilus parainfluenzae Meningitis in an Adult Post-COVID-19 Infection

**DOI:** 10.7759/cureus.57076

**Published:** 2024-03-27

**Authors:** Shirisha Pasula

**Affiliations:** 1 Infectious Diseases, Northeastern Ohio Infectious Diseases, Youngstown, USA

**Keywords:** sinusitis, post-covid-19 complications, hemophilus parainfluenzae, gram negative meningitis, meningitis

## Abstract

*Hemophilus parainfluenzae* is a gram-negative coccobacillus that usually inhabits the respiratory tract. It is a causative agent of meningitis, usually in children. The author presents a case of a 34-year-old woman presented with fever, neck stiffness, and headache, two weeks after the diagnosis of coronavirus disease 2019 (COVID-19) infection. Her CT scan of the head showed sinusitis. CSF analysis showed monocytic pleocytosis. CSF cultures grew *Hemophilus parainfluenzae*. The patient improved on intravenous antibiotic ceftriaxone 2 grams every 12 hours. This article also provides a brief literature review of *Hemophilus parainfluenzae* infections associated with COVID-19 infection.

## Introduction

Bacterial meningitis is an inflammation of the meninges caused by bacteria. Clinical manifestations encompass fever, neck stiffness, and altered mental status. A definitive diagnosis of meningitis is made by cerebrospinal fluid (CSF) analysis and bacterial culture [1]. Community-acquired meningitis is caused mainly by *Streptococcus pneumoniae*, *Neisseria meningitidis,* and *Hemophilus influenzae* [2]. *Listeria monocytogenes *can cause meningitis in patients, who are older than 50 years of age, neonates, and immunocompromised [2]. The mode of entry for meningitis is either contiguous spread from sinuses or CSF leak or hematogenous spread from bacteremia or endocarditis.

*Hemophilus parainfluenzae* is a gram-negative coccobacillus and fastidious organism. It is an inhabitant of the respiratory tract. It can cause upper and lower respiratory tract infections, bacteremia, endocarditis, and meningitis [3]. Hemophilus species is one of the HACEK (*Haemophilus, Aggregatibacter, Cardiobacterium, Eikenella, Kingella*) organisms that cause endocarditis in adults [4]. Meningitis from *Hemophilus parainfluenzae* is mostly reported in children [3,5]. It is unusual in adults. Here, we report a case of *Hemophilus parainfluenzae* meningitis in an adult female after coronavirus disease 2019 (COVID-19) infection.

## Case presentation

A 34-year-old Caucasian female presented to the emergency department with a fever, headache, neck pain, nausea, and vomiting for three days. Two weeks prior to the current evaluation, the patient developed nasal congestion and dry cough leading to a COVID-19 diagnosis. The symptoms resolved without requiring any medical treatment. Three days ago, she woke up with a fever and a headache, which initially improved with acetaminophen 500mg as needed. However, the fever recurred and progressed to neck pain, nausea, vomiting, and low appetite. The patient did not have any associated symptoms such as cough, shortness of breath, postnasal discharge, abdominal pain, diarrhea, or skin rash.

Past medical history includes scoliosis, for which corrective surgery was performed during adolescence. The patient has two children. One was five years old, and the other was two and a half years old. Both children were reported to be healthy. One of the children’s classrooms reported a case of hand-foot-mouth disease recently. They were vaccinated appropriately for their ages. Two weeks ago, the patient attended a wedding in a wooded location and had numerous mosquito bites. The patient did not smoke and consumed alcohol occasionally.

**Figure 1:**
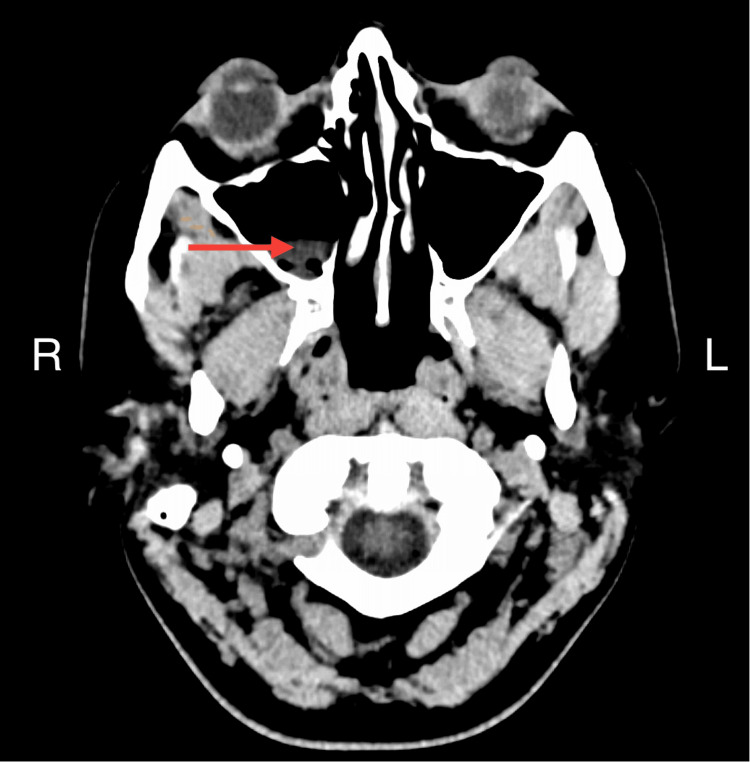
CT scan of the head The red arrow shows the right maxillary sinus with mucosal thickening suggesting sinusitis R: right, L: left

**Figure 2:**
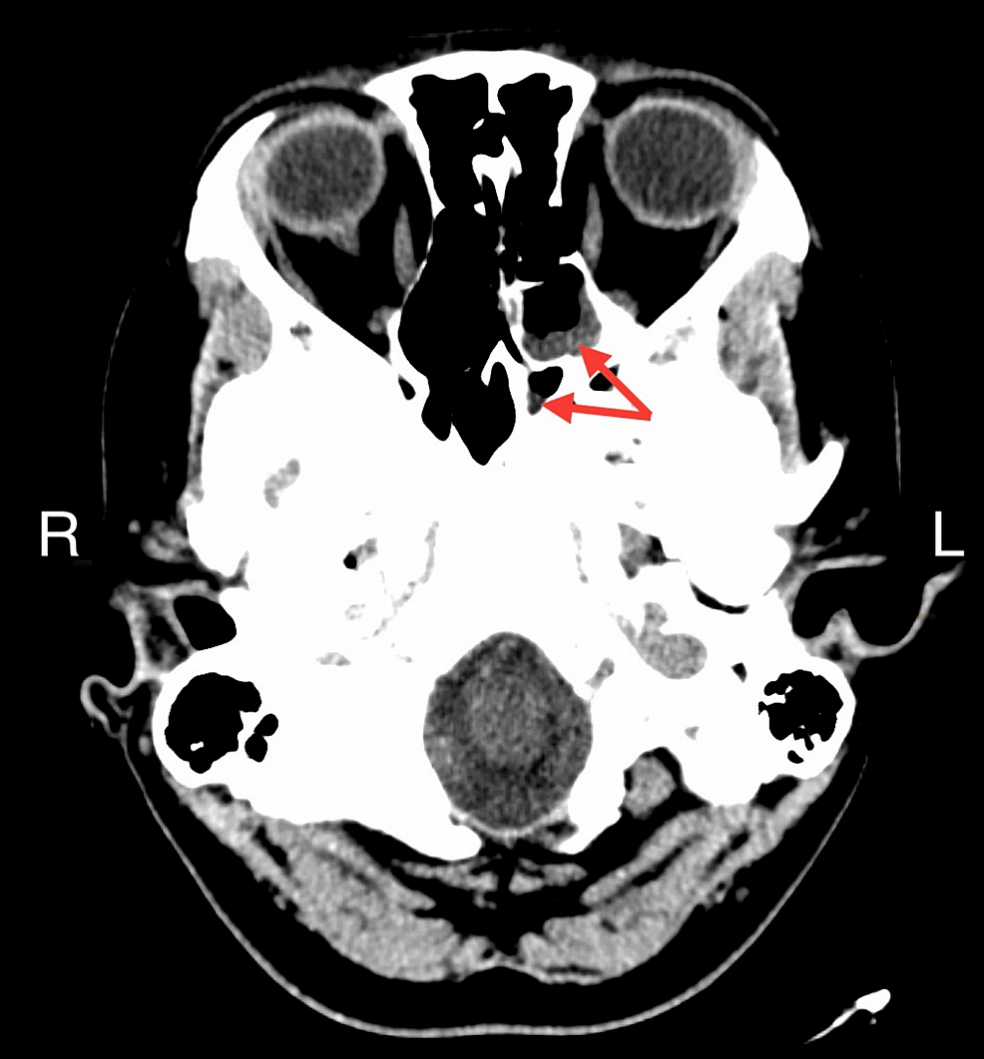
CT scan of the head Red arrows point to the left ethmoid and sphenoid sinus with mucosal thickening suggesting sinusitis.

Vital signs include blood pressure of 104/68 mmHg, pulse 89/minute, temperature 101.3 F, and respiratory rate 20/minute. On physical examination, the patient did not have neck stiffness, sinus tenderness, or neurological deficits. Auscultation revealed clear lungs. Lab studies are reported in Table 1. The chest X-ray was clear. CT head showed right maxillary, left ethmoid, and sphenoid sinusitis (Figures 1, 2). No sign of meningitis on the imaging. The patient underwent lumbar puncture and received intravenous (IV) vancomycin 1500 milligrams one dose and ceftriaxone 2 grams one dose. Cerebrospinal fluid (CSF) analysis results are in Table 1.

**Table 1:** Lab results CSF: cerebrospinal fluid; k/ul: thousands per cubic milliliter; g/dl: gram per deciliter; mg/dl: milligram per deciliter; cells/ul: cells per cubic milliliter

Lab test	Result	Reference range and units
White count	13 (high)	4.5 - 11.5 k/uL
Hemoglobin	12.5	11.5 - 15.5 g/dL
Platelet count	296	130 - 450 k/uL
Blood cultures	No growth	No growth
CSF appearance	Clear	-
CSF glucose	61	40-70 mg/dl
CSF protein	45.2 (high)	15-40 mg/dl
CSF volume	12	mL
CSF RBC	<2000	Cells/ul
CSF WBC	59 (high)	0-5 cells/ul
CSF neutrophils	41 (high)	0-6%
CSF monocytes	59 (high)	15-45%

CSF Gram stain showed many polymorphonuclear leukocytes, many mononuclear leukocytes, and no organisms. The differential diagnosis included bacterial Vs viral infection including West Nile and Lyme disease. CSF meningitis polymerase chain reaction (PCR) panel was negative. The panel includes *Escherichia coli, Hemophilus influenzae, Listeria monocytogenes, Neisseria meningitidis, Streptococcus pneumoniae, Cytomegalovirus, Enterovirus, Herpes simplex 1&2, Human herpes virus-6, Parechovirus, Varicella-zoster virus,* and* Cryptococcus neoformans/gatti*. CSF West Nile PCR and Lyme panel were negative. On day 3, one colony of growth was identified on the chocolate agar media from the CSF culture. Given it was only one colony of organisms, MALDI-TOF (Matrix Assisted Laser Desorption Ionization-Time of Flight) was employed for organism identification. The analysis identified the microorganism as *Hemophilus parainfluenzae*. Additionally, MALDI-TOF analysis also yielded information regarding the organism's susceptibility, indicating sensitivity to beta-lactam antibiotics. Antibiotic susceptibilities are not routinely performed on this organism. It was requested to be sent out to the reference laboratory. Antibiotic susceptibilities are mentioned in Table 2. She tested negative for HIV and her immunoglobulin (IgG, IgA, and IgM) levels were normal. The patient was treated with IV ceftriaxone 2 grams every 12 hours. She clinically improved and her fever resolved three days after starting treatment. Intravenous antibiotics were continued for a total of 14 days.

**Table 2:** Hemophilus parainfluenzae antibiotic susceptibilities MIC: minimum inhibitory concentration; ug/mL: micrograms/mL Susceptibility testing was performed by CLSI (Clinical and Laboratory Standards Institute) - approved broth dilution method using custom-made MIC panels.

Antibiotic	MIC (ug/mL)	Interpretation
Ampicillin	0.5	Susceptible
Ceftriaxone	0.12	Susceptible
Meropenem	<0.06	Susceptible

## Discussion

Meningitis due to *Hemophilus parainfluenzae* is uncommon in adults. Risk factors for *Hemophilus parainfluenzae* infection include respiratory comorbidities, immunocompromised status, and complement deficiencies. Having young children may have increased the likelihood of this patient acquiring the infection. It is probable that this patient initially developed sinusitis, which spread contiguously to the meninges resulting in meningitis.

Acute bacterial meningitis typically results in neutrophilic predominance in the CSF. Only around 10% of patients have lymphocytic or monocytic predominance [1]. Our patient had only 59 cells/ul of WBCs in the CSF. The differential showed that 59% of the WBC were monocytes and 41% were neutrophils.

There is an extensive body of research published on bacterial and fungal coinfections with COVID-19 [6-8]. The most common respiratory pathogens associated were *Staphylococcus aureus*, *Streptococcus pneumoniae, *and *Hemophilus influenzae* [8]. Fungal infections like aspergillosis, mucormycosis, and cryptococcal infections were also identified in patients who were critically ill from COVID-19 infection [9].

There are three cases of *Hemophilus parainfluenzae* coinfection with COVID-19 that have been published so far. Finch et al. reported a case of a young man who presented with COVID-19 pneumonitis and features of cardiogenic shock [10]. His blood culture turned positive on day 5 for *Hemophilus parainfluenza,* and he was found to have mitral valve endocarditis along with embolic brain infarcts. The patient underwent mitral valve repair and was treated with Intravenous ceftriaxone 2 grams twice daily for six weeks with excellent recovery [10]. There was another report of *Hemophilus parainfluenzae* endocarditis along with COVID-19 infection has been published by Castro et al.* *[4]. Xueting Ou et al. reported a case of a 65-year-old woman admitted with severe respiratory failure with COVID-19 pneumonia and sputum cultures also showed *Hemophilus parainfluenzae* and* Moraxella catarrhalis* [11]. No treatment information was provided.

Viral infections like COVID-19, Flu A/B, and Middle East respiratory syndrome (MERS) can result in the dysregulation of the immune system, rendering it susceptible to opportunistic infections [8]. High suspicion leading to further workup and early treatment is necessary to improve mortality.

*Hemophilus* species are usually susceptible to ampicillin but there are reports of ampicillin resistance, which can be detected by beta-lactam sensitivity testing [12]. There is no specific guidance on *Hemophilus parainfluenzae* meningitis, but Infectious Disease Society of America (IDSA)/American Heart Association (AHA) guidelines recommend third-generation cephalosporins as the recommended regimen for endocarditis and meningitis by *Hemophilus* species [1,12].

## Conclusions

This case of *Hemophilus parainfluenzae* meningitis post-COVID-19 infection supports that COVID-19 infection is a risk factor for acquiring opportunistic infections. Physicians should be vigilant for unusual presentations of infectious diseases in the post-COVID-19 period. In patients with clinical signs of meningitis, it is important not to sway solely by CSF PCR panel and wait for culture results for slow-growing organisms. Third-generation cephalosporins are the recommended first-line management for* H.* *influenzae* and *parainfluenzae* infections.
